# Decreased functional connectivity between ventral tegmental area and nucleus accumbens in Internet gaming disorder: evidence from resting state functional magnetic resonance imaging

**DOI:** 10.1186/s12993-015-0082-8

**Published:** 2015-11-18

**Authors:** Jin-Tao Zhang, Shan-Shan Ma, Sarah W. Yip, Ling-Jiao Wang, Chao Chen, Chao-Gan Yan, Lu Liu, Ben Liu, Lin-Yuan Deng, Qin-Xue Liu, Xiao-Yi Fang

**Affiliations:** State Key Laboratory of Cognitive Neuroscience and Learning, IDG/McGovern Institute for Brain Research, Beijing Normal University, Beijing, China; Center for Collaboration and Innovation in Brain and Learning Sciences, Beijing Normal University, Beijing, China; CASA Columbia, Department of Psychiatry, Yale University School of Medicine, New Haven, CT USA; Key Laboratory of Behavioral Science and Magnetic Resonance Imaging Research Center, Institute of Psychology, Chinese Academy of Sciences, Beijing, China; The Nathan Kline Institute for Psychiatric Research, Orangeburg, NY USA; Department of Child and Adolescent Psychiatry/NYU Langone Medical Center Child Study Center, New York University, New York, NY USA; Institute of Developmental Psychology, School of Psychology, Beijing Normal University, Beijing, China; Faculty of Education, Beijing Normal University, Beijing, China; Key Laboratory of Adolescent Cyber Psychology and Behavior (CCNU), Ministry of Education, Wuhan, China; Academy of Psychology and Behavior, Tianjin Normal University, Tianjin, China

**Keywords:** Internet gaming disorder, Resting-state functional connectivity, Ventral tegmental area, Nucleus accumbens, Craving

## Abstract

**Background:**

Internet gaming disorder (IGD) has become an increasing mental health problem worldwide. Decreased resting-state functional connectivity (rsFC) between the ventral tegmental area (VTA) and the nucleus accumbens (NAcc) has been found in substance use and is thought to play an important role in the development of substance addiction. However, rsFC between the VTA and NAcc in a non-substance addiction, such as IGD, has not been assessed previously. The current study aimed to investigate: (1) if individuals with IGD exhibit alterations in VTA-NAcc functional connectivity; and (2) whether VTA-NAcc functional connectivity is associated with subjective Internet craving.

**Methods:**

Thirty-five male participants with IGD and 24 healthy control (HC) individuals participated in resting-state functional magnetic resonance imaging. Regions of interest (left NAcc, right NAcc and VTA) were selected based on the literature and were defined by placing spheres centered on Talairach Daemon coordinates.

**Results:**

In comparison with HCs, individuals with IGD had significantly decreased rsFC between the VTA and right NAcc. Resting-state functional connectivity strength between the VTA and right NAcc was negatively correlated with self-reported subjective craving for the Internet.

**Conclusions:**

These results suggest possible neural functional similarities between individuals with IGD and individuals with substance addictions.

**Electronic supplementary material:**

The online version of this article (doi:10.1186/s12993-015-0082-8) contains supplementary material, which is available to authorized users.

## Background

Internet gaming disorder (IGD) is defined as a failure to control one’s impulses to excessively use online gaming. IGD results in serious negative outcomes, such as decreased physical and mental health, low academic achievement, and problems in interpersonal relationships [1, 2]. IGD is an increasing mental health problem worldwide [3], and has been highlighted as a topic deserving further study in Section III of the DSM-5 [4].

Resting-state functional connectivity (rsFC) analyses of functional magnetic resonance imaging (fMRI) data allow for the assessment of dynamic interactions between brain regions while the brain is ‘at rest’, and this methodology has increasingly been used to study the neurobiology of addictions [5–8]. Researchers have proposed that reduced mesolimbic rsFC may relate to impaired dopamine signaling which would result in a blunting of activity in regions within the reward pathway, or a reduced hedonic set point, among individuals with addictions [5, 9]. Dopaminergic (DAergic) reward pathways have been intensively studied and are associated with generation of craving and addictive behaviors [10–13]. Tomasi and Volkow have suggested that ventral tegmental area (VTA) and substantia nigra (SN) connectivity may be a potential biomarker for DAergic dysfunction disorders [14]. Gu et al. reported decreased rsFC between the VTA and the nucleus accumbens (NAcc) in chronic cocaine users [5]. Dopamine neurons within the VTA project to the NAcc as part of the mesolimbic pathway and communication between these regions is essential for acute drug reward [13, 15] and implicated in incentive motivation [16], such as substance-related craving [17–19]. In a study of adult smokers, Hong et al. found a weakened rsFC strength between the dorsal anterior cingulate and striatum [20].

Previous studies have demonstrated that IGD individuals have alterations in resting-state patterns of brain activity involving regions responsible for reward, impulse control and sensory-motor coordination, similar with neural mechanisms underlying drug abuse [21, 22]. Several lines of evidence implicated the dopamine system in IGD. For example, Han et al. found that the Taq1A1 allelic variant of the DRD2 gene is associated with excessive playing of Internet games [23]. Increased NAcc activity in response to gaming-related cues has been reported among individuals with IGD (compared to healthy controls), and is positively correlated with self-reported craving for Internet gaming [24]. Therefore, it is possible that IGD and craving for Internet gaming may be associated with alterations in NAcc–VTA functional connectivity, as has been reported among individuals with substance addictions.

This study had two primary aims: First, to examine and compare rsFC between the NAcc and VTA using seed-based functional connectivity analyses among IGD participants and healthy comparison (HCs) participants. Second, to explore the relationship between VTA and NAcc functional connectivity and the Internet gaming-related craving. Based on the previous studies discussed above, we hypothesized that rsFC between the NAcc and VTA would be decreased among IGD participants in comparison to HC participants. In addition, we hypothesized that connectivity strength would be negatively correlated with subjective craving for internet use.

## Methods

### Participants

Four hundred and thirty-two individuals were recruited from several universities in Beijing city by online advertisement. Given the increased incidence of IGD among men versus women [25], and in order to control for any possible gender effects on neural activity, only male participants were included in this study. Based on the inclusionary/exclusionary criteria described below, thirty-five IGD subjects and 24 HC subjects were selected to participate in this study. The Ethics Committee of State Key Laboratory of Cognitive Neuroscience and Learning, Beijing Normal University approved the study. All participants provided written informed consent for the study and were compensated for their participation.

### Inclusionary criteria for IGD and HC participants

IGD participants had to meet the criteria of Internet addiction proposed by Ko and Chen [26], as assessed using the Chinese Internet Addiction Scale (CIAS) [27]. This scale consists of 26 self-report items associated with online Internet use problems, including items related to tolerance, withdrawal, compulsive Internet use, time management, interpersonal relationship problems and health problems. Each item uses a scale from ‘Strongly disagree’ (score = 1) to ‘Strongly agree’ (score = 4), and the CIAS total score is calculated by the sum of the items. The reliability and validity of the CIAS among college students has been demonstrated previously [27]. According to Ko et al. [26], people who have a score of 67 or greater meet criteria for Internet addiction. Other inclusion criteria for IGD participants were that their primary Internet activity was Internet gaming, that the majority of their time spent on the Internet (i.e., more than 50 % of hours of Internet use) involved Internet gaming [28, 29], and that the time spent on Internet gaming per week was greater than 14 h (i.e., average of more than 2 h per day) [30].

Inclusion criteria for HC participants were a score less than 60 on the CIAS (an optimal cutoff point for screening of Internet addiction is 63/64, according to Ko et al. [26]), and occasionally Internet gaming (or no lifetime gaming) over the history of their normal Internet use.

Exclusion criteria for all participants included a history of neurological or psychiatric disease including all other types of addiction, and current and past use of psychotropic medications. The Fagerstrom Test for Nicotine Dependence (FTND) [31] and the Michigan Alcoholism Screening Test (MAST) [32] were used to make sure all the participants were free of smoking and drinking problems. The Beck Anxiety Inventory (BAI) [33] and the Beck Depression Inventory (BDI) [34] were used to assess the participants’ current anxiety and depression state. Participants with scores of 17 or more on the BAI and of 21 or more on the BDI were excluded [35].

### Subjective craving for internet gaming

An eight-item Likert scale was adapted from the brief Questionnaire of Smoking urges (QSU-brief) [36]. All participants were asked to evaluate their craving for Internet use (for HCs) or online gaming (for IGD participants) on a 7-grade Likert scale, from “1 = I don’t want to at all” to “7 = I extremely want to”. A higher score on this scale indicates a higher craving for Internet use or online gaming. The questionnaire was administered immediately prior to scanning.

### Data acquisition

Resting-state fMRI data were collected on a 3.0T Siemens Trio scanner at Beijing Normal University. Participants were instructed to keep their head motionless and eyes open and not to think about anything systematically during the scanning. A helmet and earplugs were also used to minimize head motion and machine noise. Scanning parameters were as follows: repetition time (TR) = 2000 ms, echo time (TE) = 30 ms, flip angle (FA) = 90°, thickness = 3.5 mm, field of view (FOV) = 192 mm × 192 mm, acquisition matrix = 64 × 64, voxel size = 3.1 × 3.1 × 3.5 mm^3^, gap = 0.7 mm, NIslice = 33, time point = 200.

### Data processing

The software DPARSF Version 2.1 (Data Processing Assistant for Resting-state fMRI, http://www.restfmri.net) [37] was used to pre-process imaging data. Prior to pre-processing, the first 10 s of data were discarded for each participant due to shimming time. Slice timing correction, realignment and spatial normalization and smoothing (full-width-half-maximum, FWHM = 4 mm) were conducted. Nuisance covariates including cerebrospinal fluid signals, global mean signals, white matter signals, and six head motion parameters were regressed from the functional MR imaging data. Participants whose head motion translation values were more than 3.0 mm in any direction of x, y, z or rotation values more than 3° of any angle were excluded. The frame-wise displacement (FD) of head position [38] was not different between groups (*t* = 1.32, *p* = 0.192, Table 1). We applied a scrubbing regression to reduce the effects of head motion. Time points with FD of 0.2 or higher were assigned values of 0, and other time points were assigned values of 1. Scrubbing regression allows for the same number of time points across participants while controlling for excess head motion at specific time points [39–41].

**Table 1:** Demographic characteristics and behavioral information for IGD and HC groups

	IGD (n = 35)	HC (n = 24)	*t*/Mann–Whitney U/χ^2^
Age	22.46 ± 2.21	23.13 ± 2.09	−1.16
CIAS	76.23 ± 7.67	45.79 ± 9.61	13.51***
Time spent on Internet gaming	23.77 ± 12.01	1.07 ± 0.45^n^	−6.58 ^a,^***
Craving	30.83 ± 7.48	13.42 ± 6.82	9.10***
Anxiety	5.40 ± 4.88	3.04 ± 3.16	4.15*
Depression	7.67 ± 5.65	2.96 ± 2.99	2.25***
Alcohol use	28	18	0.21^b^
Smoking	5	0	3.75^b^
Mean FD	0.12 ± 0.04	0.11 ± 0.05	1.32

Mean ± SD are shown

Mean FD: the frame-wise displacement (FD) of head position

*CIAS* the score of Chinese Internet Addiction Scale

*** p < 0.001, * p < 0.05

^n^There were only 7 of 24 HCs playing internet games

^a^Mann–Whitney U test

^b^Chi-square test

REST version 1.8 (http://restfmri.net/forum/REST_V1.8) [42] was used for later data processing. Data were de-trended and filtered (0.01–0.08 Hz) to reduce both low-frequency drift and high-frequency physiologic noise.

**Fig. 1:**
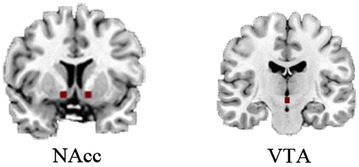
Regions of interest (ROIs) of NAcc (± 12, 8, −8, 6 mm radius) and VTA (0, −16, −7, 3 mm radius) in Talairach Daemon coordinate

Consistent with methods published by Gu et al. [5], regions of interest (ROIs) were defined by placing spheres centered on coordinates: left NAcc (Talairach Daemon coordinate: −12, 8, −8, 6 mm radius), right NAcc (Talairach Daemon coordinate: 12, 8, −8, 6 mm radius) and VTA (Talairach Daemon coordinate: 0, −16, −7, 3 mm radius) [15] (Fig. 1), which were provided by the Talairach Daemon database [43]. Then, rsFC between the three seeds was calculated by the Pearson’s correlation coefficient of each of the seed’s time courses; correlation coefficients had already been converted to Fisher’s Z values to promote the normality of data distribution.

### Data analysis

#### Group analysis

A two-sample t test was used to explore differences in the rsFC strength (Fisher Z value) between the three ROIs between the IGD and HC groups. In addition, we also compared the whole brain rsFC with each ROI as a seed, using alphasim correction with voxel-level p < 0.01 and cluster-level p < 0.05 was used (results shown in Additional file 1).

### Association with clinical variables

Correlation analysis involving rsFC strength (Fisher Z value) and the score of CIAS/craving was performed to investigate the relationship between rsFC strength and severity of IGD/craving for the Internet (for HC participants) or Internet gaming (for IGD participants) across groups and in each group separately.

## Results

Demographic characteristics and behavioral information are shown in Table 1.

### Group difference in rsFC

**Fig. 2:**
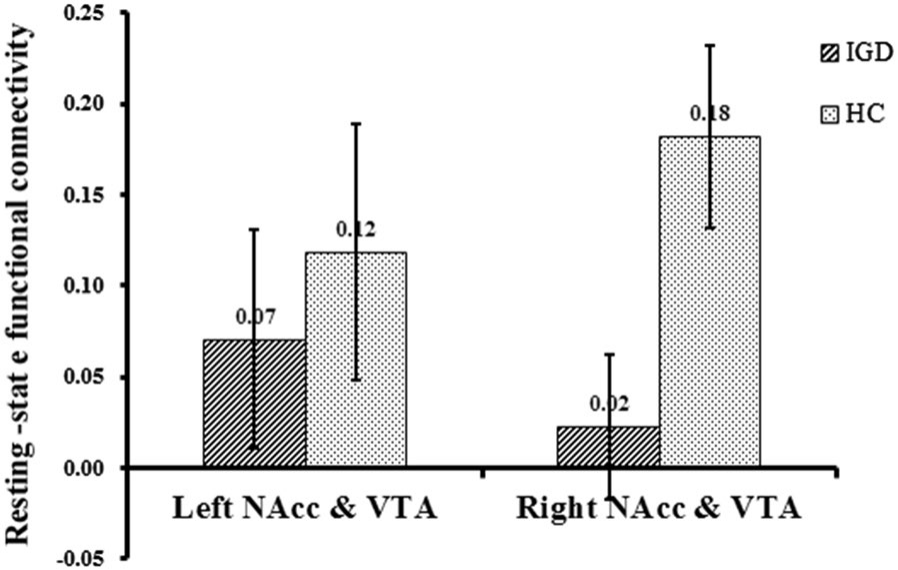
Resting-state functional connectivity (rsFC) between the VTA and left/right NAcc. The label’ Left NAcc and VTA’ refers to the rsFC strength between these two regions of interest. *Bars with oblique lines* show the mean values of rsFC between the VTA and left/right NAcc in IGD, *bars with dots* show the mean value of rsFC between the VTA and left/right NAcc in HC, *error bars* the standard errors of the mean

Findings from between-group comparisons are shown in Table 2 and Fig. 2. In comparison with HC participants, the IGD participants exhibited significantly decreased rsFC strength between the VTA and right NAcc (*p* = 0.021). By contrast, rsFC between the VTA and left NAcc was not significantly different between HCs and IGD participants (*p* = 0.591). Whole-brain between-group differences in rsFC are shown in the Additional file 1: Table S2; Figures S1, S2.

**Table 2:** Resting-state functional connectivity (rsFC, Fisher Z value) difference between IGD and HC

The rsFC strength	IGD (n = 35)	HC (n = 24)	*t* value	*p*	*d*
Left NAcc and VTA	0.07 ± 0.06	0.12 ± 0.07	−.54	.591	0.15
Right NAcc and VTA	0.02 ± 0.04	0.18 ± 0.05	−2.37*	.021	0.64

Mean ± SD are shown

Left NAcc and VTA means the rsFC strength between these two regions of interest

*p < 0.05

IGD subjects had significant higher BDI and BAI scores than HC subjects. Given concern related to the post hoc inclusion of covariates [44], we did not include these variables as covariates in our primary data analyses. Exploratory follow-up analyses including these variables yielded the following results for between-group differences in right NAcc and VTA connectivity: anxiety as covariate: *F* = 3.92, *p* = .053; depression as covariate: *F* = 1.38, *p* = .245. However, it is important to note that BDI and BAI scores were both significantly related to game-playing hours per week (r = 0.47, *p* < 0.001; r = .38, *p* = 0.003) and CIAS scores (r = 0.49, *p* < .001; r = 0.41, *p* = .001, Additional file 1: Table S1). Therefore, including these measures as covariates may have removed variance explained by problematic Internet game-playing or IGD severity.

### The relationship between connectivity strength and severity of IGD/craving

**Fig. 3:**
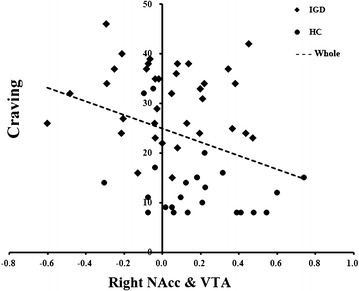
Scatterplot showing association between right NAcc–VTA functional connectivity and subject craving across groups. *Black squares* indicate the values for IGD participants, *black circles* indicate the values for HC participants. *Dotted line* represents the best-fit regressions for all subjects

The relationships between rsFC strength of the three regions and severity of IGD/craving for the Internet (for HC participants) or Internet gaming (for IGD participants) were assessed using correlation analyses. The results are shown in Table 3 and Fig. 3.

**Table 3:** Correlation between resting-state functional connectivity (rsFC) strengths and CIAS/craving

The rsFC strength	All participants (n = 59)	
	CIAS	Craving
Left NAcc and VTA	0.00 (*p* = 0.974)	−0.03 (*p* = 0.816)
Right NAcc and VTA	−0.30* (*p* = 0.020)	−0.32* (*p* = 0.013)

Left NAcc and VTA means the rsFC strength between these two regions of interest

*p < 0.05

In the correlation analysis, the rsFC strength between the VTA and right NAcc was significant negatively correlated with the severity of IGD (CIAS, *p* = 0.974 for VTA and left NAcc and *p* = 0.020 for VTA and right NAcc) and with craving (*p* = 0.816 for VTA and left NAcc and *p* = 0.013 for VTA and right NAcc).

We also tested the correlation between rsFC strength and severity of IGD/craving in each group separately, and found no significant correlations within either group. Comparison of r values using Fisher’s r-to-z transformation (http://vassarstats.net) indicated that r values did not differ between groups for associations with CIAS (VTA–left NAcc: Z = −0.94, *p* = 0.348; VTA–right NAcc: Z = 0.81, *p* = 0.416) or craving (VTA–left NAcc: Z = −0.40, *p* = .689; VTA–right NAcc: Z = 0.25, *p* = 0.801). See results in Additional file 1: Table S3.

## Discussion

To our knowledge, this is the first study to investigate rsFC between the VTA and NAcc among individuals with IGD versus controls, and to assess associations between rsFC and subjective craving. In comparison to HC participants, individuals with IGD had significantly decreased rsFC between the VTA and right NAcc. In addition, rsFC between these ROIs was significantly negatively associated with craving ratings for Internet-gaming.

### RsFC between the VTA and NAcc

Our findings are consistent with the decreased rsFC between the VTA and the NAcc in chronic cocaine users, reported by Gu et al. [5]. The mesolimbic dopamine system—involving connections originating in the VTA and projecting to the NAcc has been consistently implicated in the neurobiology of addictions [45, 46]. Hypothesized decreases in mesolimbic dopamine signaling may be related to impaired dopamine signaling [5] and alterations in reward learning frequently observed among individuals with addictions [47], and are consistent with theoretical models proposing blunting of reward pathways, or reduced hedonic set points, among individuals with addictions, as noted by Gu and colleagues [9].

Functional alterations in mesolimbic pathways among individuals with drug or alcohol addictions are often interpreted as a consequence of frequent, long-term exposure to an exogenous drug. However, none of the IGD participants in this study had a history of drug or alcohol problems. Therefore, it is also possible that reduced VTA–NAcc connectivity in this population may represent a vulnerability factor for the development of IGD. In addition, the strength of VTA–right NAcc was negatively correlated with the severity of IGD, suggesting this rsFC could reflect the state of IGD, consistent with the finding that VTA and SN connectivity serves as a potential biomarker for DAergic dysfunction disorders [14].

IGD participants in this study had significantly higher anxiety and depression scores than did control participants. Both anxiety and depression were significantly associated with IGD severity and after including these variables in post hoc analyses the between-group difference in VTA and right NAcc connectivity was no longer significant. Prior studies have reported elevated anxiety and depression scores among frequent Internet users [48, 49], which were positively related with problematic Internet use [50–52]. In addition, among psychological variables considered, depression has been most strongly associated with the development of IGD [53]. Thus for individuals with IGD, higher depression and anxiety might be a representative indicator of problematic Internet game-playing or IGD severity. Further studies explicitly recruiting individuals with IGD with low levels of anxiety and depression are needed to disentangle the effects of these variables on rsFC.

### The association between rsFC strengths and craving

RsFC between the bilateral NAcc and VTA was significantly negatively correlated with craving, such that individuals with greater subjective Internet-craving had decreased rsFC. This inverse association between mesolimbic rsFC and subjective craving intensities is consistent with previous studies implicating rsFC between the bilateral NAcc and VTA in substance-related craving [17–19], and can be explained by the theoretical models positing blunted DAergic transmission among individuals with both drug and behavioral addictions [54]. Within this framework, it is possible that long-time Internet gaming could impair VTA–NAcc connectivity.

There was no significant linear correlation between VTA and right NAcc connectivity and CIAS or craving among individuals with IGD. Within the context of the incentive-habit model of addiction [55], it may be argued that with increases in severity of IGD, Internet gaming may shift from a reward-directed behavior to a habitual compulsive behavior, and thus may be more closely involved in the maintenance of Internet gaming playing, rather than with craving specifically [56]. Further research assessing changes in craving processes in relation to rsFC over time among individuals with IGD is needed to explore this hypothesis.

Increased NAcc activity in response to gaming-related cues has been reported among individuals with IGD (compared to HCs), and is positively correlated with self-reported craving for Internet gaming [24]. Thus further research is needed to determine how alterations in functional connectivity might relate to cue-induced activity of the VTA and NAcc in this population.

Our findings have significant clinical implications: Craving is a central component of both substance and behavioral addictions [57–59], and is an important potential therapeutic target. Our finding of a negative association between VTA and NAcc connectivity and subjective craving suggests that interventions specifically targeting craving processes in IGD may also impact on mesolimbic connectivity, or vice versa. Our findings suggest possible overlapping pathways of substance addictions and IGD; thus effective interventions for substance dependence may also be effective for IGD. More generally, these data suggest a possible general mechanism that may exist across addictions, which could therefore be a potentially sensitive biomarker for diagnoses as well as a target for treatment.

## Conclusions and limitations

This study has several limitations. First, the cross-sectional design prevents us from determining whether alterations in rsFC are a vulnerability factor for, or a consequence of, IGD. Second, group sizes are unbalanced in our study, which may have influenced the results. Third, this study did not include female participants. While IGD is most prevalent among men, the exclusion of women limits the generalizability of our findings. In addition, we mainly focused on findings from our ROI-based connectivity analysis (rather than a whole-brain approach). However, this method was more appropriate to our research questions and allowed for comparison with other studies in substance addictions which have used VTA and NAcc ROIs [5]. We also conducted a whole-brain analyses for comparison and discussion in future studies.

The present study is the first to demonstrate decreased rsFC strength between the VTA and NAcc among individuals with IGD, in comparison to HCs. This is also the first study to report associations between VTA and NAcc connectivity and self-reported craving among individuals with IGD. These results parallel findings from substance addictions, suggesting possibly shared neurobiological substrates between IGD and substance addictions and providing additional support to the notion that IGD is a behavioral addiction.

## Additional file

10.1186/s12993-015-0082-8 Correlations between VTA-NACC connectivity and IGD severity/subjective craving within each group, and the whole-brain between-group differences in resting state functional connectivity of VTA/NACC.
