# Supplementary material for: Decreased functional connectivity between ventral tegmental area and nucleus accumbens in Internet gaming disorder: evidence from resting state functional magnetic resonance imaging
Source: Behav Brain Funct. 2015 Nov 18;11:37. doi: 10.1186/s12993-015-0082-8 (PMC4652358; doi:10.1186/s12993-015-0082-8)
Supplement: Supplementary file 1 — 10.1186/s12993-015-0082-8 Correlations between VTA-NACC connectivity and IGD severity/subjective craving within each group, and the whole-brain between-group differences in resting state functional connectivity of VTA/NACC. [file 12993_2015_82_MOESM1_ESM.docx]

**sTable1** Correlation between BDI/BAI and CIAS/ game-playing hours per week

|  | CIAS | game-playing hours per week |
| --- | --- | --- |
| BDI | .485^**^ (*P* < .001) | .38^***^ (*P* < .001) |
| BAI | .410 (*P* = .001) | .47^**^ (*P* < .001) |

**sTable 2** Group difference in resting-state functional connectivity (rsFC) across whole-brain (a positive t-value indicates IGD>HC; a negative t-value indicates IGD<HC)

| Seed | Region | Hemisphere | Cluster size | X | Y | Z | Peak t value |
| --- | --- | --- | --- | --- | --- | --- | --- |
| Left NAcc | Precentral Gyrus | R | 58 | 36 | -3 | 39 | 3.95 |
| VTA | Postcentral Gyrus /Superior Temporal Gyrus | L | 73 | -63 | -18 | 6 | -4.12 |
| Right NAcc | Inferior Frontal Gyrus | R | 167 | 60 | 24 | 15 | 5.09 |

Note. Alphasim correction with voxel-level P < 0.01 and cluster-level p<0.05.

**sTable 3** Correlation between rsFC strengths and CIAS/craving within IGD or HC

| The rsFC strength | IGD (n=35) | | HC (n=24) | | | |
| --- | --- | --- | --- | --- | --- | --- |
|  | CIAS | Craving | | | CIAS Craving | |
| Left NAcc & VTA | .09 (*p*=.607) | -.22 (*p*=.296) | | .20 (*P*=.348） | | .04 (*p*=.872) |
| Right NAcc & VTA | -.06 (*p*=.727) | -.07 (*p*=.693) | | -.13 (*P*=.553) | | -.29 (*p*=.166) |

Note. Left NAcc & VTA means the rsFC strength between these two regions of interest.

**sTable 4** Difference of rsFC between IGD and HC (no global signal regression)

| The rsFC strength | IGD(n=34) | HC (n=23) | *t* | *P* | *d* |
| --- | --- | --- | --- | --- | --- |
| Left NAcc& VTA | 0.18±0.05 | 0.23±0.05 | -.60 | .549 | 0.16 |
| Right NAcc& VTA | 0.16±0.04 | 0.29±0.04 | -1.93 | .058 | 0.52 |

Note. 1. Mean±SE are shown.

2. Left NAcc & VTA means the rsFC strength between these two regions of interest.

**sTable 5** Correlation between rsFC strengths and CIAS/craving (no global signal regression)

| The rsFC strength | All Participants (n=59) | |
| --- | --- | --- |
|  | CIAS | Craving |
| Left NAcc & VTA | -.04 (*p*=.765) | \| -.04 (*p*=.794) \| \| --- \| \| -.23(p=.082) \| |
| Right NAcc & VTA | -.23 (p=.078) | -.23 (*p*=.082) |

Note. Left NAcc & VTA means the rsFC strength between these two regions of interest.

**sTable 6** Difference of rsFC between IGD and HC (scrubbing FD≥0.5)

| The rsFC strength | IGD(n=35) | HC (n=24) | *t* | *P* | *d* |
| --- | --- | --- | --- | --- | --- |
| Left NAcc & VTA | 0.05±0.05 | 0.14±0.05 | -1.24 | .220 | 0.33 |
| Right NAcc & VTA | 0.18±0.04 | 0.18±0.05 | -2.35^*^ | .022 | 0.63 |

Note. 1. Mean±SE are shown.

2. Left NAcc & VTA means the rsFC strength between these two regions of interest.

**sTable 7** Correlation between rsFC strengths and CIAS/craving (scrubbing FD≥0.5)

| The rsFC strength | All Participants (n=59) | |
| --- | --- | --- |
|  | CIAS | Craving |
| Left NAcc & VTA | \| -.11 (*p*=.407) \| \| --- \| \| -.304(p=.019) \| | -.09 (*p*=.498) |
| Right NAcc & VTA | -.23 (*p*=.082) | -.30^*^ (*p*=.019) |

Note. Left NAcc & VTA means the rsFC strength between these two regions of interest.

sTable 8 Subjective craving questionnaire for Internet (gaming)

| Items | I don’t want to at all I extremely want to | | | | | | |
| --- | --- | --- | --- | --- | --- | --- | --- |
| 1. I have a desire for Internet (online gaming) right now. | 1 | 2 | 3 | 4 | 5 | 6 | 7 |
| 1. Nothing would be better than Internet (online gaming) right now. | 1 | 2 | 3 | 4 | 5 | 6 | 7 |
| 1. If it were possible, I probably would use Internet (play online gaming) now. | 1 | 2 | 3 | 4 | 5 | 6 | 7 |
| 1. All I want right now is surf the Internet ( online gaming). | 1 | 2 | 3 | 4 | 5 | 6 | 7 |
| 1. I have an urge for Internet use (online gaming). | 1 | 2 | 3 | 4 | 5 | 6 | 7 |
| 1. Internet (online gaming) would feel good now. | 1 | 2 | 3 | 4 | 5 | 6 | 7 |
| 1. I would do almost anything for Internet (online gaming) now. | 1 | 2 | 3 | 4 | 5 | 6 | 7 |
| 1. I am going to use Internet (play online gaming) as soon as possible. | 1 | 2 | 3 | 4 | 5 | 6 | 7 |


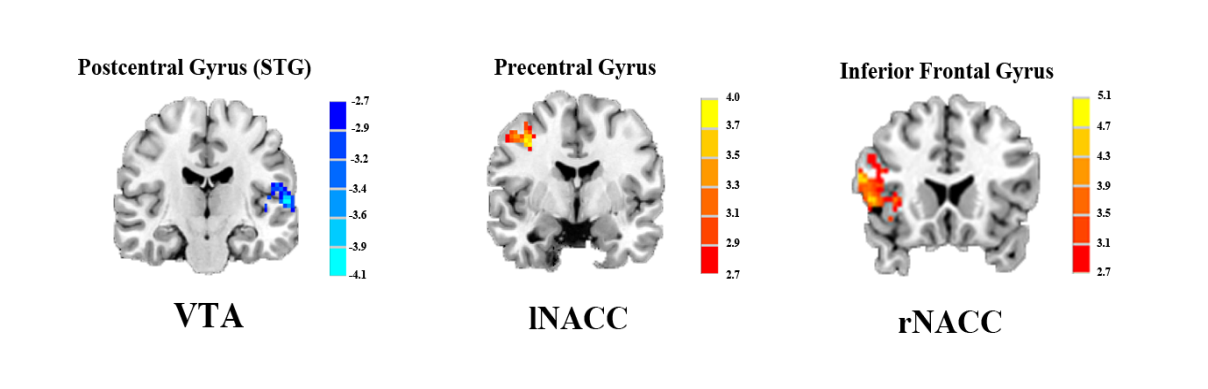


sFigure 1 Group difference in functional connectivity of VTA, lNACC, rNACC (*p*_corrected_<0.05, a positive t-value indicates IGD>HC; a negative t-value indicates IGD<HC).





sFigure 2 Group difference in resting-state functional connectivity (rsFC) across whole-brain.

VTA-Postcentral Gyrus (STG): strength of rsFC between ventral tegmental area and Postcentral Gyrus (Superior Temporal Gyrus); lNACC-Precentral: strength of rsFC between left nucleus accumbens and Precentral Gyrus; rNACC-Inferior Frontal Gyrus: strength of rsFC between right nucleus accumbens and inferior frontal gyrus. Bars with oblique line: the mean values of rsFC in IGD; Bars with dot: the mean values of rsFC in HC; Error bars indicated the standard errors of the mean.
